# Loneliness and biomarkers of brain pathology in people with subjective cognitive decline

**DOI:** 10.1038/s41598-025-95411-1

**Published:** 2025-04-02

**Authors:** Mariola Zapater-Fajarí, Isabel Crespo-Sanmiguel, Nira Cedres, Therese Rydberg Sterner, Lina Rydén, Simona Sacuiu, Margda Waern, Anna Zettergren, Henrik Zetterberg, Kaj Blennow, Silke Kern, Vanesa Hidalgo, Alicia Salvador, Eric Westman, Ingmar Skoog, Daniel Ferreira

**Affiliations:** 1 Division of Clinical Geriatrics, Center for Alzheimer Research, Department of Neurobiology, Care Sciences, and Society, Karolinska Institutet, Stockholm, Sweden; 2 Laboratory of Cognitive Social Neuroscience, Department of Psychobiology and IDOCAL, University of Valencia, Valencia, Spain; 3 Facultad de Ciencias de La Salud, Universidad Fernando Pessoa Canarias, Las Palmas, España; 4 Department of Psychology, Sensory Cognitive Interaction Laboratory (SCI-Lab), Stockholm University, Stockholm, Sweden; 5 Centre for Ageing and Health, The University of Gothenburg, Gothenburg, Sweden; 6 Neuropsychiatric Epidemiology Unit, Department of Psychiatry and Neurochemistry, Institute of Neuroscience and Physiology, Sahlgrenska Academy, University of Gothenburg, Gothenburg, Sweden; 7 Region Västra Götaland, Sahlgrenska University Hospital, Clinic for Psychiatry, Cognition and Old Age Psychiatry, Gothenburg, Sweden; 8 Region Västra Götaland,Sahlgrenska University Hospital , Psychosis Department, Gothenburg, Sweden; 9 Clinical Neurochemistry Laboratory, Sahlgrenska University Hospital, Mölndal, Sweden; 10 IIS Aragón, Department of Psychology and Sociology, Area of Psychobiology, University of Zaragoza, Teruel, Spain; 11 Spanish National Network for Research in Mental Health CIBERSAM, 28029 Madrid, Spain; 12 Department of Neuroimaging, Centre for Neuroimaging Sciences, Institute of Psychiatry, Psychology and Neuroscience, King’s College London, London, UK

**Keywords:** Lonely, Depressive symptomatology, Alzheimer’s disease biomarkers, Cerebrovascular disease, Subjective cognitive impairment, Risk factors, Psychology, Cognitive neuroscience

## Abstract

Loneliness is a neuropsychiatric symptom that has been associated with cognitive impairment and dementia. We aimed to investigate whether depressive symptomatology and biomarkers of Alzheimer’s disease (AD) and cerebrovascular disease (CVD) are associated with loneliness. Secondly, we aimed to investigate whether loneliness, depressive symptomatology, and biomarkers of AD and CVD are associated with subjective cognitive decline (SCD). We included 215 cognitively unimpaired participants (70 y/o) with cerebrospinal fluid biomarkers, magnetic resonance imaging, and questionnaires for loneliness, depressive symptomatology, and SCD. For aim 1, our findings showed that CVD and depressive symptomatology were the most relevant measures to discriminate people with loneliness. For aim 2, a random forest classification model showed that loneliness contributed to discriminate individuals with SCD, but logistic regression showed that its partial predictive effect was non-significant when depressive symptomatology and AD biomarkers were included in the models. We conclude that loneliness is associated with SCD, CVD, and depressive symptomatology. Given the complex interplay between loneliness, depressive symptomatology, and SCD, more research is needed to fully clarify the unique role of each neuropsychiatric symptom in relation to biomarkers of brain pathology.

## Introduction

The experience of subjective cognitive complaints (SCCs) endorsed by older people has gained attention in recent years. The self-reported perception of cognitive decline that cannot be detected on objective cognitive testing defines the clinical diagnosis of subjective cognitive decline (SCD)^1^. SCD is thought to reflect the earliest signs of Alzheimer’s Disease (AD), with several studies showing that SCD is related to future development of dementia^1–3^. SCD has been associated with AD pathological changes, including amyloid-beta plaques, tau neurofibrillary tangles, and neurodegeneration^4–6^. In addition, several studies have also demonstrated an association of SCD with cerebrovascular disease (CVD)^6–8^. These findings suggest that SCD could be an early indicator of various brain pathologies that may be clinically detectable before the onset of objective cognitive impairment.

Recently, the focus has been on whether SCD not only reflects brain pathologies but also neuropsychiatric conditions such as depression. Indeed, SCD and depressive symptomatology often co-occur, most frequently later in life^1,8,9^. Another neuropsychiatric symptom that is gaining attention is loneliness. Recent publications have suggested that loneliness increases during ageing and could be associated with a greater risk of dementia, adverse health outcomes, and mortality^10,11^. Given that loneliness and depressive mood are closely related^12,13^, some studies have investigated the role of loneliness in SCD, suggesting that memory complaints are more frequent in older people with loneliness^14–19^. This finding highlights the role of loneliness in individuals at risk of dementia. Furthermore, it is estimated that around a third of people with dementia feel lonely^20^. However, the literature for the association between loneliness and biomarkers of brain pathology is still limited. Two previous studies showed that loneliness is related to cortical amyloid burden and higher tau binding in positron emission tomography (PET) in brain areas of early tau accumulation in older adults^21,22^.

Another common finding of aging, SCD, and dementia is the presence of cerebrovascular disease (CVD), which can be assessed on magnetic resonance imaging (MRI). An in-vivo longitudinal study showed an association between loneliness and increased volume of brain white matter signal abnormalities (WMSA)^23^, an established MRI marker of CVD that is associated with an increased risk of cognitive impairment and dementia^24^. However, a post-mortem study showed that ante-mortem feelings of loneliness were not related to CVD or AD pathology at autopsy^25^. Therefore, it is important to elucidate the association of loneliness with SCD and biomarkers of AD and CVD, in-vivo.

The main goal of this study was to investigate loneliness in the context of SCD, depressive symptomatology, and biomarkers of AD and CVD in a population-based cohort of cognitively unimpaired individuals. Firstly, we investigated whether depressive symptomatology and biomarkers of AD and CVD were associated with loneliness. AD biomarkers were assessed through amyloid-beta and phosphorylated tau levels in the cerebrospinal fluid (CSF) and CVD was assessed through WMSA on MRI. We hypothesized that both depressive symptomatology and biomarkers of AD and CVD would be associated with loneliness^12,13,21–23^. Secondly, we investigated whether loneliness, depressive symptomatology, and biomarkers of AD and CVD were associated with SCD. We pursued to ascertain whether the potential association between loneliness and SCD was independent of depressive symptomatology, due to the known association of depressive symptomatology with both loneliness and SCD^8,13^. We hypothesized that both loneliness and AD and CVD biomarkers would be associated with SCD, independently of depressive symptomatology^4–7,14^. In addition, there is an emerging interest in describing the role of specific subjective cognitive complaints, e.g. memory *vs.* non-memory complaints^26,27^. Different cognitive complaints may reflect different syndromic and biomarker profiles^28,29^. Therefore, we investigated whether our findings would differ for memory and concentration complaints. We hypothesized that subjective memory complaints would be associated with AD pathology, as memory impairment is common in typical AD, while concentration complaints would be more associated with CVD pathology, because difficulties in concentration have been observed in people with CVD^27,28^.

## Results

### Key characteristics of the cohort

Table 1 shows the key characteristics of the cohort (N = 215). All individuals were 70 years old and 82.2% were born in Sweden. Fifty-three percent of the individuals were women and the average number of years of education was 13 ± 4 years. 32% of the participants were *APOE* ε4 carriers. Regarding biomarkers, 31% of the individuals had abnormal Aβ42/40 ratio levels, 6% had abnormal p-tau levels, and 14% had a high WMSA volume. A total of 30 individuals (14%) endorsed feelings of loneliness ranging from rarely (20%), sometimes (70%), and very often (10%). A total of 23 individuals (11%) endorsed concentration complaints, 119 individuals (55%) endorsed memory complaints, and 87 individuals (41%) endorsed neither memory nor concentration complaints. Following the SCD-I criteria for assessing depression, all individuals were within the normal range of depressive symptomatology when using clinical cut points derived from the MADRS-10 (M = 3, SD = 3.67), while 6 participants had mild depressive symptomatology, and 1 participant had moderate depressive symptomatology^30^.

**Table 1:** Characteristics of the cohort.

	Total sample (N = 215)	Loneliness group (N = 30)	Non-loneliness control group (N = 185)	*t (p)/X*^*2*^*(p)*
Age (years)	70.54 ± 0.26	70.56 ± 0.26	70.54 ± 0.27	0.358 (0.720)
Sex %Women (N)	53 (114)	80 (24)	48.6 (90)	10.186 (0.001)
Without a partner %(N)	26.2 (56)	70 (21)	19 (35)	34.696 (< 0.001)
Living alone %(N)	34 (73)	70 (21)	28.1 (52)	20.201 (< 0.001)
Years of education	13.30 ± 4.10	14.53 ± 4.64	13.13 ± 3.98	1754 (0.081)
Income (SEK/month after tax)	17,300.73 ± 7852.91	15,518.27 ± 5252.47	17,607.64 ± 8192.29	− 1255 (0.211)
MMSE	29.19 ± 1.07	29.23 ± 1.01	29.18 ± 1.08	0.235 (0.815)
SCD memory complaints %(N)	55.3 (119)	60 (18)	54.6 (101)	0.305 (0.581)
SCD concentration complaints %(N)	10.7 (23)	26.7 (8)	8.1 (15)	9.307 (0.002)
Depressive symptomatology M (SD)	3 ± 3.67	7.03 ± 5.19	2.34 ± 2.89	4.833 (< 0.001)
Aβ42/40 ratio abnormal levels %(N)	30.8 (66)	43.3 (13)	28.8 (53)	2.553 (0.110)
p-Tau abnormal levels %(N)	6 (13)	3.3 (1)	6.5 (12)	0.452 (0.501)
WMSA (high volume)%(N)	14.4 (31)	23.3 (7)	13 (24)	2.245 (0.134)
*APOE* ε4 carriers %(N)	32.2 (68)	30 (9)	32.6 (59)	0.079 (0.778)

*MMSE* Mini Mental State Examination, *SCD* Subjective Cognitive Decline, *Aβ42/40* Amyloid-beta 42/40 ratio, *p-tau* phosphorylated tau, *WMSA* White matter signal abnormalities, *APOE-ε4* participants with at least one apolipoprotein ε4 allele; Depressive symptomatology was assessed with the MADRS-10 scale; *χ*^*2*^ Chi-square.

### Aim 1: the association of depressive symptomatology and AD and CVD biomarkers with loneliness

Univariate analysis showed that those endorsing loneliness feelings included more women and individuals who did not have a partner, lived alone, had concentration complaints, and had higher depressive symptomatology (Table 1).

**Fig. 1:**
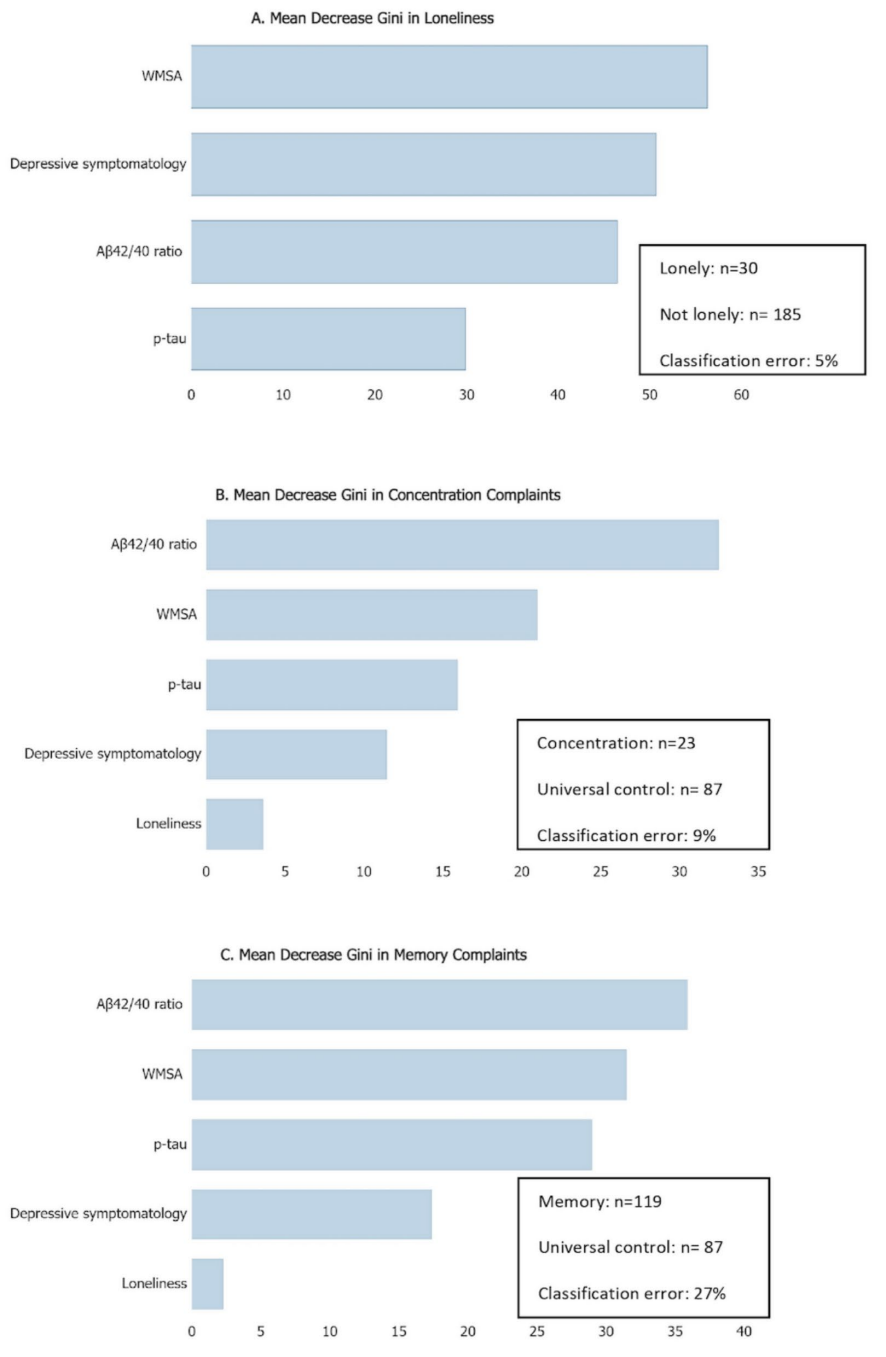
Contribution of AD and CVD biomarkers and depressive symptomatology towards predicting loneliness (**A**); and loneliness, AD and CVD biomarkers, and depressive symptomatology towards predicting SCD (**B,C**). Data represent the mean decrease in the Gini (mdGini) parameter. *Aβ42/40* Amyloid-beta 42/40 ratio, *p-tau* phosphorylated tau, *WMSA* White matter signal abnormalities, *SCD* Subjective Cognitive Decline; Depressive symptomatology was assessed with the MADRS-9 scale.

The multivariate random forest analysis with the loneliness group as the dichotomous outcome and depressive symptomatology, Aβ42/40 ratio, p-tau, and WMSA as predictors (continuous) achieved an excellent classificatory performance (classification error: 5%, Fig. 1). WMSA was the most important variable in the classification (mdGini = 56), followed by depressive symptomatology, the Aβ42/40 ratio, and p-tau (all with a mdGini < 50) (Fig. 1a).

To investigate the partial effect of each predictor in predicting loneliness, we conducted a logistic regression model (Table 2). Prior to logistic regression, the factorial analyses reduced the predictors down to two factors that explained 55% of the total variance. Factor 1 included Aβ42/40 ratio, p-tau and WMSA, and Factor 2 included depressive symptomatology (29% and 26% of the explained variance, respectively). The logistic regression model showed that the depressive symptomatology-factor was associated with an increased odds ratio for endorsing loneliness feelings (Table 2). In contrast, the biomarkers-factor including Aβ42/40 ratio, p-tau, and WMSA was not statistically significant in the prediction of loneliness.

**Table 2:** Logistic regression with loneliness as the dichotomous outcome.

Predictors: Factor 1 (Aβ42/40 ratio, p-tau, and WMSA) and Factor 2 (depressive symptomatology)					
χ^2^(2) = 31.811, *p* < 0.001, *R*^2^ = 0.249 (Nagelkerke)					
Predictor	OR	Wald	B	SE	p
Factor 1	0.970	0.021	− 0.031	0.211	0.883
Factor 2	2.965	25.152	1.087	0.217	< 0.001

*Aβ42/40* Amyloid-beta 42/40 ratio, *p-tau* phosphorylated tau, *WMSA* White matter signal abnormalities; Depressive symptomatology was assessed with the MADRS-9 scale, *OR* odds ratio, *SE* standard error.

### Aim 2: the association of loneliness, depressive symptomatology, AD and CVD biomarkers with concentration and memory complaints

#### Concentration complaints

The multivariate random forest analysis with concentration complaints as the outcome variable (dichotomous: SCD concentration complaints group *vs.* non-complaints control group) and loneliness, Aβ42/40 ratio, p-tau, WMSA, and depressive symptomatology as predictors had an excellent classificatory performance (classification error: 9%). Aβ42/40 ratio was the most important variable in the classification (mdGini = 32), followed by WMSA, p-tau, depressive symptomatology, and loneliness (all mdGini < 21) (Fig. 1b). Next, we conducted a logistic regression model to investigate the partial effect of each of these predictors in predicting concentration complaints. We set up a simplified logistic regression model with loneliness and the two factors from the factorial analysis described above (Factor 1: Aβ42/40 ratio, p-tau, and WMSA; and Factor 2: depressive symptomatology), as predictors. Concentration complaints group was included as the dichotomous outcome. This logistic regression model showed that the depressive symptomatology-factor was associated with an increased odds ratio for concentration complaints (*p* = 0.004), but loneliness and the biomarkers-Factor 1 were not significant predictors (*p* = 0.078 and *p* = 0.081, respectively, see Table 3).

**Table 3:** Logistic regression with loneliness, Factor 1 (Aβ42/40 ratio, p-tau, and WMSA) and Factor 2 (depressive symptomatology) as predictors.

Outcome: SCD concentration complaints					
χ^2^(3) = 21.107, *p* < 0.001, *R*^2^ = 0.274 (Nagelkerke)					
Predictor	OR	Wald	B	SE	p
Factor 1	1.545	3.035	0.435	0.250	0.081
Factor 2	2.422	8.368	0.885	0.306	0.004
loneliness	3.285	3.105	0.1.189	0.675	0.078

Outcome: SCD memory complaints					
χ^2^(3) = 3.201, *p* = 0.362, *R*^2^ = 0.021 (Nagelkerke)					
Predictor	OR	Wald	B	SE	p
Factor 1	1.097	0.420	0.092	0.142	0.517
Factor 2	1.187	1.157	0.171	0.159	0.282
loneliness	1.448	0.587	0.370	0.483	0.444

SCD group as the dichotomous outcome.

*Aβ42/40* Amyloid-beta 42/40 ratio, *p-tau* phosphorylated tau, *WMSA* White matter signal abnormalities; Depressive symptomatology was assessed with the MADRS-9 scale, *SCD* subjective cognitive decline, *OR* odds ratio, *SE* standard error.

#### Memory complaints

The multivariate random forest analysis with memory complaints as the outcome variable (dichotomous: SCD memory complaints group *vs.* non-complaints control group) and loneliness, Aβ42/40 ratio, p-tau, WMSA, and depressive symptomatology as predictors had a good classificatory performance (classification error: 27%). Aβ42/40 ratio was the most important variable in the classification (mdGini = 36), followed by WMSA, p-tau, depressive symptomatology, and loneliness (all mdGini < 31) (Fig. 1c). Next, we conducted a logistic regression model to investigate the partial effect of each of these predictors in predicting memory complaints. The logistic regression model included loneliness and the two factors from the factorial analysis described above as predictors, and the memory complaints group as the dichotomous outcome. This logistic regression model for memory complaints was not statistically significant (Table 3).

## Discussion

This study investigated the association of depressive symptomatology and biomarkers of AD and CVD with loneliness and SCD. We found that increased brain pathology and, especially, increased WMSA contributed to the discrimination of individuals with loneliness. While models testing for combined effects (i.e. random forest) showed contributions from all investigated variables in the prediction of loneliness, when testing for partial effects (i.e. logistic regression) only depressive symptomatology was a significant predictor of loneliness. We observed similar results when predicting concentration and memory complaints.

In our study, biomarkers of AD and CVD showed a contribution towards predicting the presence of loneliness in models testing for combined effects. Specifically, CVD biomarkers of WMSA showed the highest contribution towards predicting the presence of loneliness. This result is in line with a previous longitudinal study that showed that loneliness was associated with an increased volume of WMSA over time^23^. The mechanisms underlying the association between loneliness and CVD biomarkers could be multi-faceted. On the one hand, it could be that loneliness is a consequence of the disease (e.g., CVD), that manifests as a downstream symptom. Specifically, CVD and other pathologies could be affecting neural networks related to socioemotional processing, leading to symptoms of loneliness, as suggested by d’Oleire Uquillas et al.^21^ and Duan et al.^23^. On the other hand, loneliness could rather be a risk factor and thus be one of the multiple contributors towards neurodegeneration. Specifically, loneliness is related to impaired social skills^31^, and smaller gray matter volume in brain areas related to processing of social information^32^. Therefore, lonely people could have less developed neural networks underlying social processing, which would confer lower cognitive and brain reserve and increase the vulnerability of those brain networks. This means that lonely people would have a lower capacity to compensate for common age-related brain pathologies such as CVD. Our current study contributes to the field by suggesting that CVD seems to have a stronger association with loneliness than AD biomarkers, but the causal association between loneliness and CVD biomarkers should be elucidated in future studies.

Another contribution of our study is the finding that the association between WMSA and loneliness was not significant when depressive symptomatology was also included in models testing for partial effects. This suggests that loneliness and depressive symptomatology may share most of their variance with CVD. This result differs from previous studies suggesting that brain pathology in preclinical stages of AD or CVD is associated with loneliness, independently of depression^21–23^. There are some reasons that could explain this discrepant finding. Firstly, differences in the age of participants across studies could be influencing the results because AD pathology increases with age^33^. The mean age in the studies by d’Oleire Uquillas et al.^21^ and Donovan et al.^22^ was 76 years (range from 68 to 89 years). Therefore, most of the participants in the cited previous studies were older than our participants, who were all 70 years old, and may thus have a higher frequency of AD pathology than our current cohort. Moreover, considering that the entorhinal cortex is an early site of tau neurofibrillary tangle accumulation^34^, the association between tau and loneliness reported by d’Oleire Uquillas et al.^21^ could be due to the sensitivity of the regional PET measure used in their study, in contrast to the global measure of CSF p-tau used in our current study. Further, the scale used for depression symptomatology could also partly explain the discrepant results. While all three previous studies used a scale specific for the geriatric population (Geriatric Depression Scale, GDS), the MADRS in our study is appropriate for the adult population at large. The MADRS is regarded as advantageous at capturing a broad range of symptoms perhaps with higher sensitivity for subclinical symptoms, while it may be less weighted towards capturing changes very specific to the geriatric population. Additionally, although the level of depressive symptomatology in all three previous studies was similar to that in our study, Donovan et al.^22^ reported that 8% of their participants surpassed the GDS threshold for clinical depression, whereas none of our participants surpassed that threshold as postulated by the SCD-I^1^. Our MADRS scores thus just represent variability in subclinical depressive symptomatology in our cohort. All these findings together could suggest that at age 70 and perhaps below that age, loneliness may be more strongly associated with emotional factors (e.g., subclinical depressive symptomatology), whereas at ages older than 70, loneliness could be more strongly associated with biomarkers of neurodegenerative disease and less associated with depressive symptomatology. Similarly, the SCD-I has postulated that SCD may be related to emotional factors below the age of 60, while its association with neurodegenerative disease increases above the age of 60^1^. Further research is needed to address the role of loneliness and depressive symptomatology as potential factors related to CVD. The fact that loneliness was predicted by biomarkers of brain pathology in addition to depressive symptomatology in our models testing for combined effects (i.e., random forest) opens the door to questions about disease mechanisms and causal associations between brain pathology and loneliness. The cohort in our study is rather healthy, and we cannot exclude that a higher level of neuropathology or loneliness may be necessary to capture the associations reported in previous studies, when we used models testing for partial effects (i.e., logistic regression).

Other relevant symptom or condition emerging at the earliest stage of neurodegenerative diseases is SCD. Hence, we investigated the association of loneliness with SCD operationalized as cognitive complaints in concentration and memory domains. In models testing for combined effects, we found that loneliness, depressive symptomatology, and biomarkers of AD and CVD contributed to both concentration and memory complaints. Some previous studies investigated the association between loneliness and SCD^14–19^, suggesting that loneliness is associated with a higher frequency of memory complaints. In our current study, we confirmed that loneliness contributed to memory complaints. In addition, we expanded the previous literature by demonstrating that loneliness is a less important predictor of complaints than depressive symptomatology and biomarkers of AD and CVD. The models for combined effects showed that WMSA, amyloid-beta, and p-tau biomarkers were the most important variables in classifying complaint groups, followed by depressive symptomatology and loneliness. This finding is in line with our recent studies showing associations of depressive symptomatology and biomarkers of neurodegeneration and CVD with SCD^7,8,29^. We also showed that different complaints may reflect different underlying pathologies^28^. In our current study, the models for partial effects showed that depressive symptomatology was the only statistically significant predictor for concentration complaints, while our model for memory complaints was not statistically significant. This supports the findings from Diaz-Galvan et al.^28^, suggesting different associations of complaints with biomarkers and depressive symptomatology. To summarize these findings, our study suggests that the association of loneliness with SCD is not independent of depressive symptomatology. Together with previous work demonstrating that depressive symptomatology and brain pathology are two independent factors related to SCD^8,29^, the data suggest that depressive symptomatology and feelings of loneliness may share aetiology, likely not directly related with AD and CVD pathology, in SCD individuals. Future research should continue to disentangle the association between depressive symptomatology, loneliness, brain pathology, and SCD, separately at younger and older ages.

Our study has some limitations. We investigated associations using cross-sectional data, so we cannot draw conclusions about causality in our findings. The tau biomarker was based on CSF samples (p-tau levels), whereas tau PET can provide regional information and tau uptake in entorhinal cortex may be more sensitive to capture associations as compared with the global CSF p-tau measure used in our study. The design of our cohort with all participants being 70 years old makes it difficult to extrapolate the results to other ages, although this feature in our study possibly increases the homogeneity of our sample. Finally, we used self-rated scales for depressive symptomatology, loneliness, and SCD. As it is common practice^8,29^, we relied on a global measure of depressive symptoms. Future research should consider the distinct features assessed by the MADRS depression scale and explore how specific depressive symptoms measured by the MADRS are associated with loneliness and SCD. Regarding loneliness, measures of loneliness based on a single item are widely used and validated^35^, but their sensitivity to detecting associations with biomarkers may be reduced compared with more extended or indirect measures of loneliness. Further, the negative connotations associated with the word loneliness in direct items could lead people who feel lonely to not endorse loneliness due to stigma^36^. In our study, women endorsed more loneliness than men and this could be because men are more likely to respond to an indirect question about loneliness as opposed to a direct question^37^. Regarding SCD, although single questions of memory and concentration as opposed to detailed questionnaires may be less sensitive to SCD, the SCD-I has reported that dichotomous items are widely used, and our study was able to capture several significant associations. Future research should expand current methods to provide broad assessments of loneliness and SCD, for comparison.

In conclusion, our data suggest that loneliness is associated with biomarkers of brain pathology, particularly CVD. However, depressive symptomatology was of greater importance than loneliness in the prediction of SCD. Given the complex interplay between loneliness and depressive symptomatology, future research should continue to clarify their unique associations with biomarkers of brain pathology and their role in the context of subjective cognitive complaints.

## Materials and methods

### Participants

The sample is derived from the Gothenburg H70 Birth Cohort Studies, which is a population-based study of 1203 seventy-year-old participants born in 1944, conducted from 2014 to 2016 in Gothenburg (Sweden). Full details on tests and procedures are described in Rydberg Sterner et al.^38^. For the current study, we selected the 297 individuals who had available CSF biomarkers in combination with an MRI scan that included T1-weighted sequence.

Inclusion criteria for the current study were in accordance with the leading international SCD initiative (SCD-I) working group^1,9^, as follows:

I. Normal cognition: Dementia was excluded based on a clinical diagnosis of dementia using DSM-III-R criteria, a Mini-Mental State examination (MMSE) score < 24, or a Clinical Dementia Rating > 0.5. Mild cognitive impairment (MCI) was excluded according to the criteria proposed by Jak et al.^39^ and Molinuevo et al.^40^, which are based on a comprehensive neuropsychological protocol using age-, sex-, and education-adjusted norms. Participants were classified as having MCI if at least one of the following two criteria were met:
  - Impaired scores (< 16 percentile) on two tests in at least one of the following four cognitive domains: *Memory*, assessed with Thurstone’s Picture Memory 10-word list, and remembering 12 objects; *Processing speed/executive function*, assessed with the Digit Span Forward and Backward test and the Figure Logic of the Synonyms, Reasoning, and Block design test (SRB 2); *Language*, assessed with a semantic verbal task (animals); and *Visuospatial abilities,* assessed with Block Design (Koh’s Block Test).
b. Impaired scores (< 16 percentile) on three independent tests in three out of the four cognitive domains covered by the neuropsychological protocol (memory, processing speed/executive function, language, and visuospatial abilities).

When criterion ‘a’ could not be met because the domain was evaluated by one test, criterion ‘b’ was considered. Although Jak et al.^39^ and Molinuevo et al.^40^ formulated their proposals based on the minus one standard deviation (− 1SD) cut point, we opted for the 16th percentile (which reflects − 1SD on the normal gaussian curve), due to the asymmetrical distribution of some of the neuropsychological test data in our cohort.

II. No large infarcts or tumors on brain MRI according to a neuroradiologist and no history of stroke or transient ischemic attack.
III. No medical history of neurological or psychiatric disorders (e.g., major depression), systemic diseases, or head trauma.
IV. No history of substance or alcohol abuse, based on a clinical interview, and a score of < 20 on the alcohol use disorder identification test (AUDIT)^41^.

From the initial 297 participants, 79 were excluded because they failed to satisfy the inclusion criteria as follows: 45 due to dementia or MCI, 12 due to clinically abnormal MRI, 16 due to psychiatric and neurological disorders, and 6 due to drugs/alcohol abuse. In addition, three participants were excluded due to missing data on loneliness, SCD, or civil status. Therefore, the final sample in the current study included 215 participants.

### Loneliness

A self-perceived feeling of loneliness was assessed with the question “Do you feel lonely?” from the Comprehensive Psychopathological Rating Scale (CPRS) semi-structured interview^42^. The question is rated on a four-point Likert scale from 1 (never) to 4 (very often). Responses were dichotomized into a “non-loneliness group” (response 1) and a “loneliness group” (responses 2–4) for statistical analyses. This classification reproduced the approach for CPRS items of cognitive complaints in a previous study^29^, see next section for further details).

### Subjective cognitive decline (SCD)

SCD was operationalized as in a previous study^29^. Briefly, we used two questions from the CPRS semi-structured interview^42^, which cover past month subjective cognitive complaints of memory and concentration. Complaints referred to self-perceived difficulties in memory and concentration, as compared with previous ability. Both memory and concentration complaints were rated on a seven-point Likert scale ranging from 0 (no difficulties) to 6 (severe difficulties), with intermediate options. The presence of subjective complaints was determined by the cut-off point of ≥ 2: participants who scored ≥ 2 on memory complaints were classified as having SCD in memory (SCD memory complaints group), and those who scored ≥ 2 on concentration complaints were classified as having SCD in concentration (SCD concentration complaints group). Participants who scored ≤ 1 on both CPRS questions were classified as not having SCD (non-complaints control group). We favored the dichotomous SCD variable instead of the original ordinal variable due to the nature of our statistical analyses (see below).

### Depressive symptomatology

Depressive symptomatology was assessed using *the Montgomery*-*Åsberg Depression Rating Scale* (MADRS-10)^43^, which was derived from the CPRS semi-structured interview. Items are scored on a seven-point Likert scale (ranging from 0 to 6), with higher scores indicating a higher degree of depressive symptomatology. We used the MADRS-10 to categorize the cohort. However, for the main analyses we removed the concentration item from the MADRS-10 to avoid circularity with the definition of the SCD concentration complaints group. This provided a MADRS version based on 9 items (referred to as the MADRS-9 in this article).

### AD biomarkers and APOE ε4 genotype

For AD biomarkers, we used the CSF amyloid-beta 42/40 (Aβ42/40) ratio to assess amyloid-beta pathology and the phosphorylated tau (p-tau) biomarker to assess tau neurofibrillary tangle pathology^44^. Methods for CSF sampling and analysis are fully detailed elsewhere^38^. CSF Aβ42/40 and p-tau biomarkers were treated as continuous variables in main statistical analyses. For the characterization of the sample, the cut-off proposed by Samuelsson et al.^45^ was used to dichotomize CSF Aβ42/40 ratio and p-tau biomarker levels into normal and abnormal: CSF Aβ42/40 ratio ≤ 0.082 and p-tau ≥ 80 pg/mL. To characterize the sample, the *APOE* ε4 genotype was determined using the KASPar PCR (polymerase chain reaction) SNP (single nucleotide polymorphisms) genotyping (LGC Genomics, Hoddesdon, Herts, UK), as described in Skoog et al.^46^. Participants with at least one *APOE* ε4 allele were classified as *APOE* ε4 carriers.

### Magnetic resonance imaging biomarkers of CVD

We assessed hypointense WMSA as a proxy for CVD, following previous studies that investigated WMSA in SCD^6,7^. MRI scans were acquired in a 3.0 T Philips Achieva system (Philips Medical Systems). For assessment of hypointense WMSA, we used a three-dimensional T1-weighted Turbo Field Echo (TFE) sequence (repetition time = 7.2 ms, echo time = 3.2 ms, flip angle = 9°, number of slices = 160, matrix size = 250 × 250 mm, field of view = 256 × 256, slice thickness = 1.0 mm).

Hypointense WMSA were automatically segmented with FreeSurfer 6.0.0. Briefly, The T1-weighted images were processed with the FreeSurfer 6.0.0 image analyses suite (http://surfer.nmr.mgh.harvard.edu/). FreeSurfer detects white matter hypointensities and automatically labels them using a probabilistic procedure^47^. The sensitivity of this procedure in assessing white matter damage has been demonstrated in both healthy individuals and AD patients^48,49^. WMSA volumes in milliliters (ml) were adjusted by the total intracranial volume (TIV), also obtained from FreeSurfer. This adjustment was performed by dividing the WMSA volume by the TIV of each participant^50^, and TIV-adjusted WMSA measures were used for statistical analyses.

Following Cedres et al.^51^, we classified WMSA into low and high hypointense WMSA volume using the cut-off value of 0.00321, which was developed in the same cohort as the one used in the current study. This cut-off value for hypointense WMSA resembles low and high Fazekas visual rating scale WMSA^52^. Henceforth, when we refer to WMSA, we are referring to hypointense WMSA volume. This variable was treated as a continuous parameter in the main analyses, but for the characterization of the sample, the variable was categorized as high and low as in Cedres et al.^51^, to describe the overall load of CVD. All MRI data were managed and processed through the Hive DB system^53^.

### Statistical analyses

The Pearson’s Chi-square test was used to investigate group differences (loneliness vs non-loneliness groups) across categorical variables, including sex, marital and living status, memory, and concentration complaints, *APOE* ε4 genotype, and the dichotomized measures of Aβ42/40 ratio, p-tau, and WMSA. The Student’s t-test was used for group differences (loneliness vs non-loneliness groups) when variables were continuous, including age, years of education, income, MMSE, and depressive symptomatology. Box-Cox transformations were performed for continuous variables that did not follow a normal distribution (Aβ42/40 ratio, p-tau, and WMSA)^54^.

In order to address the study’s Aims 1 and 2, we designed a statistical approach based on two types of predictive models: random forest and logistic regression. While random forest assesses the contribution of multiple variables in predicting an outcome (combined effects), logistic regression assesses how different independent variables explain partial variance of an outcome (partial effects)^55^. In particular, random forest classification analyses were performed to investigate the contribution of biomarkers and depressive symptomatology (and loneliness in Aim 2) towards a dichotomous outcome (loneliness in the study’s Aim 1 and SCD in Aim 2). These random forest models were performed using the oversampling approach for unbalanced groups. We assessed the variables’ importance by using the mean decrease in the Gini (mdGini) parameter, which reflects the decrease in the mean of the model’s discriminative classification capacity when a predictor is excluded from the model. To address the study’s Aim 1, we performed a random forest model with depressive symptomatology and AD and CVD biomarkers as predictors and loneliness as a dichotomous outcome. For the study’s Aim 2, we performed two separate random forest models, one for concentration complaints and one for memory complaints (with loneliness, depressive symptomatology, AD and CVD biomarkers as predictors and SCD group as a dichotomous outcome). Finally, we performed logistic regression analysis to investigate the partial effect of each predictor (AD and CVD biomarkers, depressive symptomatology, and loneliness) on a dichotomous outcome (loneliness in Aim 1 and SCD in Aim 2). Logistic regression was preceded by a factorial analysis to reduce the number of predictors due to limited statistical power in analyses involving the loneliness group (n = 30) and the concentration complaints group (n = 23). The factorial analysis was based on the continuous variables of depressive symptomatology, Aβ42/40 ratio, p-tau, and WMSA, with a direct oblimin rotation. We inverted inverse variables, which means that for all variables included in the factorial analysis, higher scores reflect more pathological levels of the measure.

All statistical analyses were performed using SPSS v.27 (IBM Statistics, Chicago, IL, USA) and the R programming language (R, version 3.5; R Foundation for Statistical Computing, Vienna, Austria). All *p* values were two-tailed, and the level of significance was set at *p* < 0.05.

## Data Availability

Anonymized data are available from the corresponding author on reasonable request.
